# On Dysmenorrhœa and Other Uterine Affections in Connection with Derangement of the Assimilating Functions

**Published:** 1844-07-01

**Authors:** 


### ON DYSMENORRHŒA AND OTHER UTERINE AFFECTIONS IN CONNEXION WITH DERANGEMENT OF THE ASSIMILATING FUNCTIONS. By *Edward Rigby*, M.D. Physician to the General Lying-in Hospital, &c. &c. 8vo. pp. 138. Renshaw. London, 1844.

IN a short and modest preface, Dr. Rigby invites a more careful attention to these uterine derangements than the generality of practitioners are disposed to give them. He thinks they are too often looked upon as local affections, when they are really dependent on the general health. He fears that some of the symptoms he has described, and the remedies he has proposed, may appear fanciful; but requests his readers to suspend their judgments until they give a fair trial to both. Dr. R. has had ample experience in these matters, having enjoyed the privilege of attending the Uterine Cases of St. Thomas's and Bartholomew's Hospitals.

The first chapter, consisting of 36 pages, is dedicated to various observations on those numerous disorders resulting from impaired digestion, imperfect assimilation, bad chylification, and vitiated secretions. In this chapter many authors are quoted; but Dr. Prout's works furnish the chief materials. There is also some speculation in this chapter, and a great deal of transcendental humorology, far in advance of practical medicine of the present day.

The second chapter comes nearer the specific subject of the treatise—namely, to "uterine affections intimately connected with, and, in a great measure dependent on, derangements of the assimilating processes." These are to be looked upon (and they are looked upon by all rational practitioners) as merely "the local phenomena of a general condition of the system." This general condition, our author thinks, is mostly dependent on a rheumatic or gouty diathesis.

"In applying to these affections the term uterine rheumatic gout, I wish it to be understood that I do so in default of any more appropriate term; but as this expression renders my meaning tolerably intelligible, I shall be satisfied to use it for the present." 37.

These uterine affections are chiefly of a congestive or inflammatory nature, attended with local vascular excitement, more or less acute, and exhibiting the chief features of inflammation, as heat, swelling, redness, and pain—or of a chronic form, with venous engorgements, turgescence, and hardness, and ultimately change of structure.

"The first form is more sudden in its attacks and recessions, more erratic in its movements; the latter more gradual, but fixing on the part with a firmer hold, and relinquishing it with proportional difficulty. The acute form is usually seen in connexion with dysmenorrhœal attacks, or with the uterine excitement which is generally observed in such cases at the half-way time between the menstrual periods. The other is mostly attended by chronic leucorrheal discharge, and chronic or subacute inflammation of the cervix uteri, followed by induration and organic disease. It may also be observed, that if not arrested by proper treatment, the acute sooner or later passes into the chronic form, in which case they may be looked upon as different stages of the same disease, and therefore brought under the same description. It may, however, be stated, that the majority of these affections are of a chronic or subacute nature; whether so originally, or from the circumstance that the change from the acute to the chronic form frequently takes place at an early period of the disease, is not very easy to determine." 38.

These symptoms our author has generally found to be connected with obstinate dyspepsy, and occasionally accompanied by rheumatic gout, ending in dysmenorrhœa.

Dr. R. is unable to determine the precise circumstances that determine the formation of those fibrinous exudations that are occasionally thrown off in dysmenorrhœa. There is generally in such cases some inflammatory action in a neighbouring part, as the ovary.

"If the ovary be the part affected, the patient suffers much pain in one or both groins, increased on pressure, or by putting the integuments of the part on the stretch in assuming the erect posture. It is greatly aggravated at each menstrual period, coming on for one or more days before the appearance of the discharge, and attended with much pain, throbbing, and sense of heat and swelling in the part, and generally accompanied by considerable irritation of the bladder or rectum. These are the ordinary phænomena which attend a paroxysm of this disease, but accompanied, as is seen, with symptoms of more than ordinary local congestion, and attended with rheumatic or gouty symptoms in other parts. In the more sub-acute or chronic form, the precursory stage appears to consist of that series of changes produced by increasing derangement of the assimilating functions by which the circulation gradually becomes vitiated, and local disease appears as the product of general disorder. The bowels are torpid, requiring medicine to render them sufficiently active, the evacuations are unhealthy and offensive, the tongue is furred, there is a disagreeable taste in the mouth, the breath is impure, the appetite is capricious and variable; food even in small quantity produces much troublesome distension and flatulence, the urine, is generally loaded with lithic deposits, the skin is dry and rough or moist and clammy, the muscles are flabby, the limbs ache on the slightest exertion, the extremities are cold, the face is pale and sallow, there is a bilious head-ache across the forehead and eyes, and much depression of mind." 41.

In such cases the vagina often feels full, swollen, and like a nostril stuffed by a cold; being very sensitive to the touch, rendering the motion of a carriage or even walking painful. The disease is remarkably uncertain in its attacks—going off and coming on very suddenly.

"On examination, the labia and nymphæ are usually found swollen and flabby, and copiously moistened with a thick, creamy, albuminous discharge. Occasionally, however, they are hot and turgid, the vagina is in a state of soft flabby tumefaction, its parietes in close contact with each other, and its calibre much diminished by the swelling. The mucous membrane is swollen, and shows evident marks of venous congestion; it is everywhere thickly covered with the above-mentioned white or yellowish-white discharge, and not unfrequently the canal is so exquisitely sensitive as to render the introduction of the finger very painful, and sometimes even impossible. In the earlier periods of the disease the uterus does not always appear to be the seat of much suffering during the menstrual periods, the os, cervix and inferior portion feel somewhat swollen and tense, but not particularly tender; how it is in this respect *during* a menstrual period, especially when complicated with dysmenorrhœa, I have not had the opportunity of judging, but a state of more or less vascular and nervous excitement may be presumed to exist then." 43.

One remarkable phenomenon, which we have occasionally observed in these cases, is a discharge of air from the vagina, apparently secreted from its mucous surface. The urine shews strong indications of the gouty diathesis, being loaded with the lithates. The specific gravity is greater than usual, and urea is in excess. The patients are usually hæmorrhoidally disposed. The mucous secretion of the rectum is augmented, and the fæces, if firm, are covered by this secretion. Flatus is very apt to collect in the rectum, and be discharged.

"The most prominent feature in the above enumeration of symptoms is the congested state of the pelvic vessels, which, although of transitory character at first, soon becomes more and more permanent, until at length that state of sub-acute or chronic inflammation is established which sooner or later must end in organic disease. The constitutional symptoms become greatly aggravated, the chylopoietic functions are nearly suspended; emaciation and great debility follow, and the face assumes the cachectic, cadaverous appearance, which is generally looked upon as indicating structural mischief. The local symptoms resemble those of chronic leucorrhœa, with inflammation of the cervix uteri. The pain of the back becomes more decided, and is less relieved by assuming the recumbent posture; the bearing-down when she is erect is increased; severe pain is produced by sitting down upon a hard seat and by pressing up the perineum against the inflamed os and cervix uteri, which being swollen and heavy from congestion, descend lower into the pelvis than usual. From a similar reason the evacuation of fæces, especially when the bowels are somewhat constipated, is attended with pain nearly in the same direction, the rectum during their passage pressing against the os and cervix uteri. Upon examination this part will generally be found hot, engorged, throbbing and tender to the touch of the finger; there is more or less leucorrhœa, which, although it may have been creamy and albuminous in the commencement, is apt after a while, to become watery and discoloured. Slight movement, and even assuming the erect posture, or the warmth produced by sitting on a soft-cushioned seat, are sufficient to bring a considerable aggravation of her sufferings. The catamenia become more scanty and lose their natural colour; at length they cease, apparently more from the depressed state of the system being no longer able to afford this periodical evacuation than from functional inability on the part of the uterus to secrete it. Darting pains, of an altogether different character to what she has yet perceived, now make their appearance; at first but few in number and only at long intervals, but by degrees they increase in severity and frequency; the discharge becomes more watery, the os and cervix uteri more painful and indurated, the general health more broken down, the stomach more irritable; in fact, symptoms threatening the commencement of scirrhus are but too distinctly established." 48.

The urine, in time, undergoes great change. Its specific gravity decreases—it becomes pale—and soon shews albumen—consisting of little else than water and serum of the blood, the function of the kidneys being nearly suspended. The train of symptoms is not, of course, always identical with the above account. They vary from time to time.

"In a large proportion of these cases of uterine gout there have been co-existing evidence of the gouty or rheumatic-gouty diathesis in other parts. Many patients have had inflammation and swelling of the knees, ankles, and also the smaller joints of the fingers and toes; others have suffered on some previous occasion from rheumatic fever, from which they dated all their complaints. In some the local symptoms have been arrested, but they continued to suffer from frequent flushings and sense of burning heat, both in the extremities as well as in the trunk, (particularly in the abdomen,) intense headaches, and considerable derangement of the stomach, liver and kidneys. I think I may say, that in almost every instance the pains of the limbs are a very constant symptom, and, in some instances, form a prominent feature in the patient's sufferings." 52.

But it is now time to turn from symptomatology and pathology to therapeutics. As the local affections are no more than part of a general diathesis, so constitutional treatment offers the only chance of cure. The remedial process is necessarily slow, and the patience of the sufferer is often worn out before the remedies have had sufficient time for their operation. The uterus, from its extensive sympathies, soon becomes the cause of various affections of other parts, though not the primary seat of disease itself. "It is upon the stomach, liver, and bowels that we must make our early attacks; for, until their functions have been duly regulated, little hope can be expected of a favourable progress." Our first step, then, must be to rouse these organs into greater activity.

"One active dose of calomel, of from five to eight grains, followed by a mild purge the next morning of rhubarb and magnesia, &c. will frequently produce a degree of general relief which could scarcely be credited. The tongue is cleaned, the breath has become pure, the colour improved, the limbs active and free from pain, the extremities warm, and a marked relief to the pain, tension and bearing down in the pelvis, which before had rendered her almost helpless." 55.

Leeches to the rectum Dr. R. considers more effectual than to the vagina itself, and greatly facilitate the action of alterative and laxative medicines. The first application of leeches to the hæmorrhoidal vessels is generally not so effective as the subsequent applications.

"The sense of congestion and fulness about the pelvis, the bearing down pain of the uterus, the throbbing and sense of swelling about the rectum and vagina, the peculiar and painful closed-up feel of this latter canal, and the inability to move about or even sit down without pain, all disappear, and leave the patient for a time entirely freed from her sufferings." 59.

The time of application ought to be just before the expected catamenia, or equi-distant between the two periods. If the pain be seated about the cervix or os uteri, Dr. Locock's tube is a very useful instrument in facilitating the application. The vagina may be syringed with decoct. pap. alb. and if there be much discharge, the liq. plumb. diacet. may be added. If the passage be very irritable, oil and liq. Batlei may be applied by means of a camel's hair pencil. After the dose of calomel, its action may be sustained by blue-pill or Plummer's pill at night, with a magnesian saline aperient in the morning, while nitro-muriatic acids may be exhibited internally, with or without vegetable bitters.

"The attention of the practitioner must now be devoted to the more specific treatment of the case. If the circulation be plethoric and strong, the urine scanty, high-coloured, with considerable excess of lithic acid and lithates, colchicum in the form of the acetous extract, with extract of hop or henbane, may be given at night, or night and morning, and some mild saline with sp. ætheris nitrici occasionally during the day.

"The salines, as recommended by Dr. Prout, are well worthy of attention; they not only diminish the disposition to the formation of lithic acid during the processes of primary assimilation, but allay the irritable state of the digestive organs, and the urine becomes increased in quantity and more healthy in its characters." 62.

The diet should, of course, be simple and the hours regular. Animal food once a day is quite sufficient. Ablutions, at the temperature of the atmosphere, will generally be borne, and frictions afterwards will be very beneficial.

When the disease proceeds from the acute to the chronic form, the methodus medendi must be modified. We are unable to afford space for more than one other extract.

"Where the disease assumes the rheumatic or rheumatic-gouty character, we usually find it associated with less power of general circulation, and with local symptoms of less active character. Guaiacum and iodine are valuable remedies in these affections, either separately or combined. The tinctura guaiaci ammoniata may be taken in milk night and morning; or 10 grains of pulv. guaiaci and of magnes. carb. every morning, and from 2 to 5 grs. of potass. iod. with extract of hop or henbane at night; or if it be deemed unnecessary to use the guaiacum, the potass. iod. may be given two or three times a day in sarsaparilla with liq. potassæ, and the bowels regulated by an alterative or laxative pill at night; or, if it be desirable to promote diaphoresis, by a dose of Dover's powder.

"There are few remedies which keep up a healthy action of the liver so well as the taraxacum, especially when preceded by a dose or two of mercurial medicine. In most of the affections under consideration, where it is important to maintain this function in due activity, and yet where the *constant* use of mercurials is highly inexpedient, taraxacum becomes a valuable adjunct. It is prepared under a variety of forms, but I prefer the extract as being the most certain and convenient; half a tea-spoonful at night, dissolved in a little warm milk, forms a by no means disagreeable cocoa-like drink; or it may be taken with milk and lime-water if necessary. Besides its ordinary effect on the liver, and therefore indirectly upon the bowels by supplying them with healthy bile, I have reason to think that it also acts upon the skin like sarsaparilla, and for this purpose may sometimes be advantageously combined with it." 69.

A good many cases are narrated by our author, in illustration of the foregoing principles; but these we need not introduce here. We have taken much pains to concentrate the chief points of theory and practice in this volume, which is full of valuable materials, and is evidently the result of close observation, ample experience, and sound judgment. Dr. Rigby's work is one that we can most conscientiously recommend to all classes of our professional brethren.

